# Supplementary material for: A Likelihood Approach for Real-Time Calibration of Stochastic Compartmental Epidemic Models
Source: PLoS Comput Biol. 2017 Jan 17;13(1):e1005257. doi: 10.1371/journal.pcbi.1005257 (PMC5240920; doi:10.1371/journal.pcbi.1005257)
Supplement: S1 File — (TAR.GZ) [file pcbi.1005257.s014.tar.gz › HSPH_Online-SI-Revision/Additions/MSS11/n100-extreme-SEITR_noisy/n100-extreme-SEITR_noisy_NEW_summary.pdf]

peak -8	R0	$1/\Theta_2$	num week	inf	inflog	newly	newlylog	Reff	AttackRate
mean	32.6	41.6	102.6	94.	0.9	448.6	1.1	32.7	38.5
median	27.9	35.1	100.	85.6	1.	92.1	1.2	27.9	39.7
Quantile .1	23.3	25.1	99.	59.4	0.4	57.5	0.6	23.1	22.3
Quantile .9	42.5	81.	108.	147.5	1.4	817.5	1.5	43.	46.9
45	46	4							
length	50								

peak -4	R0	$1/\Theta_2$	num week	inf	inflog	newly	newlylog	Reff	AttackRate
mean	30.2	37.1	100.6	39.9	0.2	18.2	0.1	32.3	14.6
median	19.3	38.8	100.	40.3	0.2	14.5	0.1	19.9	13.4
Quantile .1	9.6	13.9	98.	15.2	0.1	4.9	0.	10.2	3.5
Quantile .9	60.5	56.2	108.	54.6	0.4	37.7	0.2	66.2	24.3
27	10	3							
length	50								

peak	R0	$1/\Theta_2$	num week	inf	inflog	newly	newlylog	Reff	AttackRate
mean	34.	29.9	91.	31.9	0.2	14.5	0.1	197.6	9.3
median	21.4	25.9	100.	28.8	0.1	14.5	0.1	168.7	6.8
Quantile .1	4.1	4.3	50.	5.	0.	5.6	0.	110.6	3.6
Quantile .9	70.9	51.7	100.	55.6	0.3	22.5	0.1	315.7	17.2
0	0	1							
length	50								

peak +4	R0	$1/\Theta_2$	num week	inf	inflog	newly	newlylog	Reff	AttackRate
mean	40.3	23.9	105.3	38.5	0.1	18.4	0.1	563.9	6.
median	29.7	19.1	100.	28.1	0.1	12.9	0.1	473.3	5.6
Quantile .1	7.2	2.5	50.	2.1	0.	4.4	0.	137.3	1.7
Quantile .9	90.3	51.3	100.	72.5	0.3	34.	0.2	1111.2	8.4
0	0	1							
length	50								

peak +8	R0	$1/\Theta_2$	num week	inf	inflog	newly	newlylog	Reff	AttackRate
mean	39.6	21.2	105.	93.8	0.2	64.2	0.2	658.8	5.8
median	29.7	18.6	100.	46.5	0.2	35.3	0.1	595.2	5.6
Quantile .1	7.2	2.5	50.	9.	0.	5.2	0.	134.9	3.5
Quantile .9	83.4	46.	100.	146.8	0.5	104.2	0.3	1268.6	7.5
0	0	1							
length	50								
